# A new virtue of phantom MRI data: explaining variance in human participant data

**DOI:** 10.12688/f1000research.24544.1

**Published:** 2020-09-14

**Authors:** Christopher P. Cheng, Yaroslav O. Halchenko

**Affiliations:** 1 Dartmouth College, Hanover, NH, 03755, USA; 2 Department of Psychological and Brain Sciences, Dartmouth College, Hanover, NH, 03755, USA

**Keywords:** Neuroimaging, seasonal variation, MRI QA, MRI, reproducibility

## Abstract

**Background: **Magnetic resonance imaging (MRI) is an important yet complex data acquisition technology for studying the brain. MRI signals can be affected by many factors and many sources of variance are often simply attributed to “noise”. Unexplained variance in MRI data hinders the statistical power of MRI studies and affects their reproducibility. We hypothesized that it would be possible to use phantom data as a proxy of scanner characteristics with a simplistic model of seasonal variation to explain some variance in human MRI data.

**Methods: **We used MRI data from human participants collected in several studies, as well as phantom data collected weekly for scanner quality assurance (QA) purposes. From phantom data we identified the variables most likely to explain variance in acquired data and assessed their statistical significance by using them to model signal-to-noise ratio (SNR), a fundamental MRI QA metric. We then included phantom data SNR in the models of morphometric measures obtained from human anatomical MRI data from the same scanner.

**Results: **Phantom SNR and seasonal variation, after multiple comparisons correction, were statistically significant predictors of the volume of gray brain matter. However, a sweep over 16 other brain matter areas and types revealed no statistically significant predictors among phantom SNR or seasonal variables after multiple comparison correction.

**Conclusions: **Seasonal variation and phantom SNR may be important factors to account for in MRI studies. Our results show weak support that seasonal variations are primarily caused by biological human factors instead of scanner performance variation. The phantom QA metric and scanning parameters are useful for more than just QA. Using QA metrics, scanning parameters, and seasonal variation data can help account for some variance in MRI studies, thus making them more powerful and reproducible.

## Introduction

Magnetic resonance imaging (MRI) is an important data acquisition technology used to unravel the mysteries of the brain, and it is very complex. The exact constitution of MRI signals is not entirely known since it could also potentially be affected by factors such as temperature and humidity variations across seasons ^1^. Notably, a study by Meyer *et al.* noted that seasonal variations may not even correspond to the four seasons, indicating a complicated relationship between environmental factors and brain functions ^2^; thus, whether or not this variance in MRI is due to scanner effects or biological causes is unclear. This unexplained variance in MRI data hinders the statistical power of MRI studies and affects their reproducibility.

MRI quality assurance (QA) metrics are indicators of the condition of the scanner at the time of a given scan, and are used for quality control in MRI centers ^3^. In cases of significant deviation from the norm, MRI personnel look into resolving underlying hardware or software issues. Otherwise, QA results are not used for anything else, and not shared alongside large shared datasets, such as Human Connectome Project (HCP) ^4^ or the ABCD study where data is acquired across different scanners and potentially affected by scanner idiosyncrasies. It is typically unknown how seasonal and operational factors affect different types of scanning (on phantom and real subjects). We hypothesize that there may be a relationship between the QA metrics of a scanner (obtained on a phantom) and the characteristics of the MRI scans (on human participants), which affect the consecutive data analysis results and drawn conclusions.

The purpose of this study was to evaluate if phantom data could be used as a useful proxy for overall scanner operational characteristics that can help explain variance in real human subject data acquired using the same MRI scanner on dates nearby phantom QA scans. The first stage of this study analyzed the influence of phantom scanning parameters on signal-to-noise ratio (SNR), which is known to be a fundamental QA metric for MRI. The second stage of this study used SNRs from stage one from phantom data to model morphometric measures obtained using human MRI data.

## Methods

### Ethical statement

This study was approved by the Dartmouth Committee for the Protection of Human Subjects (CPHS 31408). Data collection in the individual studies was approved by the same committee (CPHS 17763, 28486, 29780, 21200 and 30389). All participants gave written informed consent for participation and re-analysis of data.

### Human participants

We used the data from 206 participants (261 scans) collected from October 30, 2017 to August 28, 2018 who participated in five studies ^17– 20^ of three labs at the Dartmouth Brain Imaging Center (DBIC). Participants ranged from 18–64 years of age. There were 78 male and 128 female participants. The MRI scanner used was the 3.0 Tesla Siemens MAGNETOM Prisma whole-body MRI system from Siemens Medical Solutions. Human participant and phantom data were collected using a 32-channel head coil.

### Phantom

The DBIC collects MRI QA data weekly (typically each Monday) on an agar phantom. For the purposes of this study we did not use DBIC QA estimates but carried out QA using MRIQC BIDS-App ^7^.

QA data, converted to a BIDS dataset (including original DICOM data under sourcedata/), is available as a ///dbic/QA DataLad dataset ^8^. Subject “qa” within that dataset contains data for the agar phantom used in weekly QA scans. QA scans contain a single T1 weighted anatomical image (192×256×256 matrix at 0.90×0.94×0.94mm) and two functional T2* weighted echo planar imaging (EPI) scans (80×80×30 matrix at 3.00×3.00×3.99mm with 200 volumes acquired with time of repetition (TR) of two seconds; not used in this study).

### Data preparation

All data at the DBIC is collected following the ReproIn convention on organizing and naming scanning protocols ^9^. To guarantee that the data would not contain variance caused by different conversion software versions through time, data from all phantom and human subjects was reconverted from raw DICOMs into BIDS datasets using consistent versions of ReproIn/HeuDiConv and dcm2niix ^10^. All phantom QA data was re-converted using ReproIn/HeuDiConv with dcm2niix (v1.0.20171215 (OpenJPEG build) GCC6.3.0), and human data from different studies re-converted using ReproIn/HeuDiConv (v0.5.3) with dcm2niix (v1.0.20181125 GCC6.3.0). HeuDiConv is programmed to automatically extract many acquisition and scanner operation parameters from DICOMs and place them alongside neuroimaging files in the BIDS dataset. For the purposes of this study, a subset of those parameters was selected as variables of interest for analysis based on prior knowledge regarding which variables could potentially affect the collected data (see Table 1). Furthermore, we added seasonal effects by using NumPy 1.18.4 inserting sine and cosine waves into the model with a period of one year to roughly estimate the four seasons; arguably, this was a very simplistic model due to our data’s short duration of under two years. Our data’s limited time range precluded us from using more elaborate seasonal models, and as noted in the introduction, seasonal effects may not exactly correspond to the four seasons. Still, we felt a simplistic representation of seasonal effects could help indicate the possibility of further investigation.

**Table 1.** Quality assurance metrics of interest in this study, categorically divided by interest.

Category	Variable description (variable name)
MR scanner operation characteristics (outside of operator control)	Transmission amplifier reference amplitude (TxRefAmp)
	Specific absorption rate (SAR)
	Scanner software version (SoftwareVersions)
Acquisition specifics (affected directly or indirectly by operator for any given acquisition)	Day time of acquisition (AcquisitionTime)
	Patient position in the scanner (ImageOrientationPatientDICOM, abbreviated as IOPD)
Proxy measures of scanner operation characteristics (possibly affected by all other variables)	Total signal-to-noise ratio (snr_total)

**Figure 1.**
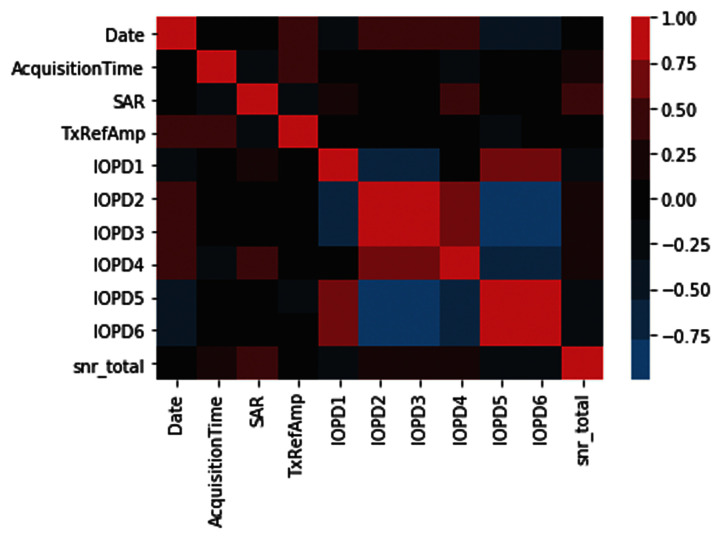
Pearson correlations between different variables on phantom MRI phantom data. Refer to Table 1 for explanation of abbreviated variables.

We used MRIQC (v0.14.2) ^7^ on both the QA phantom and the human data from October 30, 2017 to August 28, 2018. MRIQC provided us with proxy measures of scanner operation characteristics, such as total SNR for anatomicals. Figure 1 presents a correlation structure between all variables of interest for phantom MRI data visualized using Seaborn 0.10.1. DataLad ^11^ with the datalad-container extension ^12^ was used for version control of all digital objects (data, code, singularity containerized environments), and all code and shareable data were made available on GitHub (see *Data availabilit* *y* ^22^ and *Code availability* ^21^) with containers and data available from the ///con/nuisance DataLad dataset. At the moment we have concentrated on analysis of anatomical data, so only T1w images from phantom and human participants were used. 

We used DataLad to run a modified version of the simple_workflow container and script ^13^, which extracts certain segmentation statistics of the brain from real human MRI data. These include metrics relating to the accumbens area, amygdala, caudate, hippocampus, pallidum, putamen, thalamus proper, cerebrospinal fluid, and the gray and white matter in the brain. The original simple_workflow container is fully reproducible (frozen to the state of NeuroDebian as of 20170410 using nd_freeze) and uses FSL 5.0.9-3~nd80+1.

The free open source software facilitating our data preparation was Pandas 1.0.4.

### Data modeling

Ordinary least squares (OLS) regression, as implemented in StatsModels Python package (v 0.9.0) ^15^, was used to model target variables of interest. As part of the modeling, certain interdependent variables were orthogonalized to account for possible covariance (in the order presented in Figure 1; seasonal variation data was orthogonalized last) using NumPy 1.18.4. The extent to which an independent variable affects the dependent variable was assessed using a t-test for single-valued variables, and using an F-test for arrayed values (such as patient position in the scanner). Subsequently, the explanatory power of the scanning parameters and characteristics on the phantom QA metric (snr_total) as the dependent variable was evaluated.

Next, a segmentation statistic - gray brain matter on human participant data - was modeled using a proxy QA measure from phantom data (snr_total) and a scanner characteristic of the human participant scanning session (IOPD), demographics (age, gender), and seasonal effects. Gray brain matter was chosen because either gray or white brain matter were deemed likely to yield a statistically significant relationship. Because phantom QA data was acquired typically only each Monday, its value was interpolated in time to obtain values for the dates of human participants scanning. After modeling gray brain matter, other structures (such as white matter, cerebrospinal fluid, and subcortical regions) were analyzed. Our reasoning was that if gray brain matter yielded a significant result, then other brain segmentation statistics could also yield significant results, which could subsequently be investigated.

The free open source software facilitating the visualization of our model was Matplotlib 3.2.1.

## Results

### Statistical significance of variables

**Figure 2.**
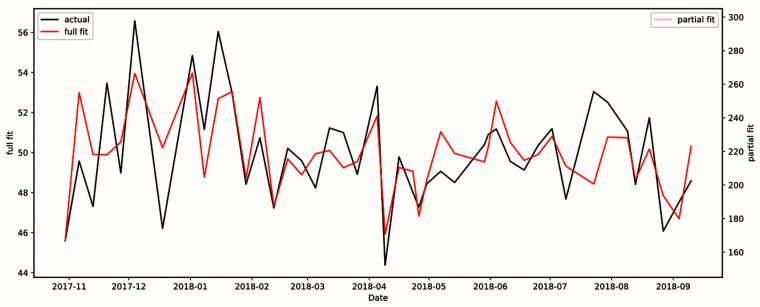
Model of total signal-to-noise ratio using scanning parameters from phantom data. R ^2^ value was 0.533, indicating scanning parameters have explanatory power.

We found that we could describe the total SNR of phantom data well with just a limited set of scanner operational characteristics. The R ^2^ value of the model shown in Figure 2 was 0.533. Multiple variables (day time of acquisition, subject position, and SAR) were statistically significant and all survived false discovery rate (FDR) correction, as shown in Table 2.

**Table 2.** Independent variables’ original and corrected p-values from the model in Figure 2. FDR, false discovery rate.

Independent variable	p-value	
	Original	FDR-corrected
Day time of acquisition	**0.019**	**0.031**
Specific absorption rate (SAR)	**0.003**	**0.017**
Transmission amplifier reference amplitude (TxRefAmp)	0.382	0.477
Patient position in the scanner (IOPD)	**0.015**	**0.031**
Seasonal variation	0.669	0.669

Given that certain scanning parameters and a QA metric were determined to have significant explanatory power in the phantom scans, we decided to proceed to model human participants’ gray brain matter volume using participants' basic demographic data, variables we found significant from phantom data (IOPD), and total SNR. Gray (or white) brain matter was deemed likely to be affected as they are two large “structures”.

After correction, the phantom’s total SNR was found to be a statistically significant (p=0.002 and FDR-corrected=0.003) predictor of gray brain matter, as shown in Table 3. Other scanning parameters and subject characteristics found significant were subject age, sex, weight, as well as seasonal variation in data. Note that the orthogonalization carried out for the model in Table 3 was carried out in the order shown from top-to-bottom.

**Table 3.** Independent variables’ original and FDR-corrected p-values from the model for gray brain matter volume ( Figure 3). FDR, false discovery rate; SNR, signal-to-noise ratio.

Independent variable	p-value	
	Original	FDR-corrected
Subject age	**0.0008**	**0.002**
Subject sex	**0.003**	**0.005**
Subject weight	**0.0000001**	**0.0000009**
Phantom’s total SNR	**0.002**	**0.003**
Subject position in scanner (IOPD)	0.056	0.056
Seasonal	**0.029**	**0.035**

After modeling gray brain matter, we modeled the other structures (background, subcortical regions, etc.), and assessed statistical significance for two independent variables of interest in each model: phantom’s total SNR, and seasonal variables. Results in Table 4 show no statistically significant results after FDR correction across structures except phantom total SNR in gray brain matter; notably, seasonal variables in gray brain matter were not significant. 

**Table 4.** Brain segmentation statistics results where bolded values are statistically significant. FDR, false discovery rate; SNR, signal-to-noise ratio; CSF, cerebrospinal fluid.

	Structure\Model	Phantom total SNR p value		Seasonal variables p value		Model R ^2^ value
		Original	FDR-corrected	Original	FDR-corrected	
**0**	Background	0.269	0.722	0.182	0.328	0.467
**1**	Left-Accumbens-area	0.981	0.981	0.879	0.973	0.104
**2**	Left-Amygdala	0.462	0.722	0.639	0.820	0.328
**3**	Left-Caudate	0.088	0.396	0.955	0.973	0.216
**4**	Left-Hippocampus	0.464	0.722	0.168	0.328	0.218
**5**	Left-Pallidum	0.563	0.722	0.160	0.328	0.520
**6**	Left-Putamen	0.464	0.722	0.087	0.225	0.262
**7**	Left-Thalamus-Proper	0.301	0.722	0.214	0.351	0.428
**8**	Right-Accumbens-area	0.569	0.722	**0.044**	0.211	0.146
**9**	Right-Amygdala	0.748	0.842	0.973	0.973	0.334
**10**	Right-Caudate	0.079	0.396	0.893	0.973	0.165
**11**	Right-Hippocampus	0.277	0.722	0.055	0.211	0.313
**12**	Right-Pallidum	0.453	0.722	0.081	0.225	0.544
**13**	Right-Putamen	0.602	0.722	0.362	0.543	0.289
**14**	Right-Thalamus-Proper	0.533	0.722	0.480	0.665	0.434
**15**	CSF	**0.031**	0.278	0.059	0.211	0.148
**16**	gray	**0.002**	**0.028**	**0.029**	0.211	0.242
**17**	white	0.862	0.912	**0.004**	0.067	0.276

### Model selection: investigation of QA metric vs seasonal effects

**Figure 3.**
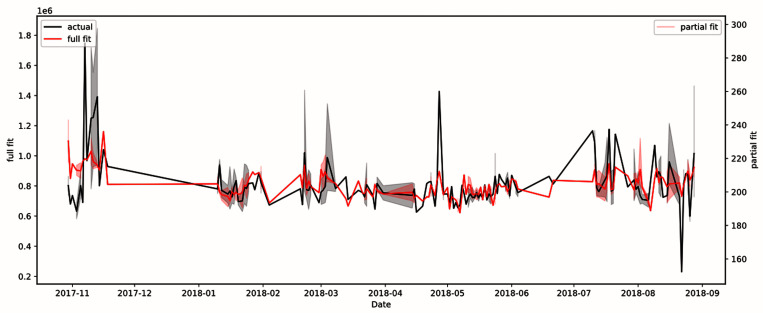
Model of gray brain matter using patient age, patient position in scanner, patient sex, patient weight, total signal-to-noise ratio, and seasonal variation. R ^2^ value was 0.242. The full fit plot (in red) shows the plot of all independent variables, whereas the partial fit plot (in pink) shows the plot of only the statistically significant variables. The black plot shows the actual fit of the real data.

Given that scanning characteristics, a QA metric and seasonal effects seem to have a statistically significant effect on gray brain matter volume estimates (see Table 3), we decided to compare the fit of the model with and without those independent variables. We did so by removing one of either the QA metric or seasonal effects from the list of independent variables in the model for gray brain matter and observing the fit of the resulting model. From the initial R ^2^ value of 0.242 (Akaike Information Criterion, AIC = 7044; Bayesian Information Criterion, BIC = 7091) with both QA metric and seasonal effects shown in Figure 3, removing the QA metric dropped the model’s R ^2^ value to 0.208 (AIC = 7054, BIC = 7097), and removing seasonal effects resulted in an R ^2^ value of 0.207 (AIC = 7052, BIC = 7092), thus showing that the QA metric and seasonal effects both have similar effects on the fit of the model. However, after removing both the QA metric and seasonal effects, the R ^2^ value drops to 0.185 (AIC = 7058, BIC = 7093), which suggests that they are complementary in terms of explanatory power.

## Discussion

Our results have indicated that the QA metric of the phantom data can be useful beyond routine monitoring of MRI scanner health. Specifically, our use case demonstrates the viability of using a QA metric to predict variance in estimates derived from human MRI scan data. Our results show also that the following scanning parameters: “patient” (in this case, phantom) position in scanner, day time of acquisition, and specific absorption rate; were statistically significant predictors of the phantom QA metric: total SNR. In turn, the phantom total SNR ratio was a statistically significant predictor of gray brain matter volume, even in the presence of actual data parameters relating to the patient such as age, sex, and weight. It seems that effects depend on the scale of data; in our data sample the uncorrected phantom QA metric provided an explanation for coarse “structures” (such as all of the gray brain matter) but failed to significantly explain any subcortical structure estimate.

Furthermore, we provide further support for the idea that seasonal variations affect human data. The initial R ^2^ value of the QA metric-human data model was 0.242; yet, by removing seasonal effects from the model, the R ^2^ value dropped to 0.207, suggesting that including seasonal effect data is useful for attributing variance in MRI data. It should be noted that the effect of the proxy scanner health metric seems to have a similar magnitude as that of seasonal variation effects, given that the removal of the QA metric from the model also results in a similar decline in the R ^2^ value to 0.208. However, we determined that this effect of seasonal variation is distinct from that from the QA metric, as can be seen by the fact that after removing both the QA metric and seasonal variation from the model, the R ^2^ value declines further to 0.185. This result indicates that both the QA metric and seasonal variations may be important variables to account for when seeking to explain variance in MRI data, given that using the MRI scanner’s phantom QA metric was not sufficient to account for all seasonal variance (at least when gray brain matter was the dependent variable). This result weakly supports the idea that seasonal variation in human data is caused by biological, rather than scanner, effects due to the fact that seasonal variation was not significant for phantom data (i.e. scanner-only) but was significant for gray brain matter in human data.

To supplement our findings on seasonal variation effects, it should be noted that our seasonal model was very simplistic and was composed of sine and cosine waves. Given that we noticed that it affected our models to some extent, we anticipate developing a more sophisticated model of seasonal effects for a more accurate model.

An unsurprising observation we made is that positioning of the patient (or phantom) in the scanner accounted for some variance. In the case of the phantom, its significance did not pass the significance threshold after FDR correction, but was very significant for human participants. Meanwhile, patient weight was a very statistically significant predictor for gray matter volume ( Table 2); conversely, the size of the phantom remained constant while SNR was affected by position in the scanner. However, prior studies have indicated that brain size strongly correlates with patient height and thus weight ^16^. This result suggests that it is still beneficial to add patient position in the scanner into the models to account for position-specific variance in addition to patient size (weight).

One interesting negative side-effect of establishing a fully reproducible pipeline, as we have done, is that we cannot share even highly compressed derivatives of the human data, such as morphometric estimates, with the subjects’ participation dates. This information could potentially be used to cross-reference with datasets where such anonymized MRI data is fully shared, albeit with their dates stripped, and thereby used to violate the confidentiality of these subjects’ data. Unfortunately, to our knowledge, no large public datasets are accompanied with phantom QA data scans from the participating sites, which made it impossible to reuse publicly available datasets.

Our use of ReproIn for “turnkey” collection of MRI data into BIDS datasets at DBIC was a highly beneficial methodology shown in our approach. An example of such a dataset is the phantom QA dataset we used in our study. The standardized structure of our dataset collection, from filenames to data format, facilitated our establishment of “meta-datasets” comprising data from multiple studies.

### Future directions

Our investigation has used phantom and human data for the period from October 30, 2017 to August 28, 2018. We are going to compare the model’s predictions on additional data (from other studies and later dates) with actual data to check the generalizability of our established models on future data and feed new data into the model to make it more robust.

As mentioned in the Methods/Phantom section, we will consider using DBIC QA estimates such as T2* weighted EPI scans instead of MRIQC estimates to evaluate the significance of other sources of QA metrics in reducing variance.

In this study we used only anatomical (T1 weighted) data. We will investigate temporal SNR, which is a QA metric only available for functional data. Functional QA metrics are an interesting area of investigation in the future, as there is some notion of functional connectivity in resting state data, and statistical estimates from GLM on task data. Functional phantom QA and other scanner characteristics could provide explanatory power to analyses.

The software we used to derive morphometric estimates of the brain could have been affected by the software used, and an investigation into the effects of conversion software (e.g., FSL) and their versions on morphometric estimates could yield valuable insights.

## Conclusions

We showed that the scanning parameters and QA metric of phantom data are useful for more than just QA. To maximize the statistical power of MRI studies, we propose using scanning parameters and a QA metric, total SNR, from an MRI scanner’s phantom data to reduce the unexplained variance that exists in MRI data.

Furthermore, we have found that our simple representation of seasonal variation can help explain gray brain matter volume in human MRI data, and deserves further investigation to determine if this effect is truly of a biological origin, as our results weakly suggest. The incorporation of seasonal variables can also help reduce variance in MRI data.

## Data availability

The human participants’ data used in this study cannot be shared, either in its anonymized form or in the form of derivative estimates of the brain structure volumes, due to the nature of the study which relies on the dates of scanning. As outlined in HIPAA, “The following identifiers of the individual or of relatives, employers, or household members of the individual must be removed to achieve the “safe harbor” method of de-identification: […](C) All elements of dates (except year) for dates directly related to the individual, including […] **admission date, discharge date**.” To apply for access to the human participant data, you may contact either of the authors ( cheng1928c@gmail.com and yoh@dartmouth.edu) and request a Data User Agreement (DUA) to fill out; upon approval, you will be granted access subject to the conditions of the DUA.

Harvard Database: Phantom MRI (Quality Assurance) Data From October 2017 to August 2018 at DBIC. https://doi.org/10.7910/DVN/PFH4FL ^22^.

This project contains the following underlying data:

- JSON files contain quality assurance metrics to be used as independent variables (AcquisitionTime, SAR, TxRefAmp, & ImageOrientationPatientDICOM) and as the dependent variable (snr_total) for logistic regression analysis
- NII.GZ files that contain a compressed version of an MRI scan in the NIfTI-1 Data Format, and is the basis from which the quality assurance metrics of the JSON file were extracted. These nii.gz files were obtained from the original DICOM files using the HeuDiConv conversion program, which you may use to validate the JSON file’s metrics.

Data are available under the terms of the Creative Commons Zero "No rights reserved" data waiver (CC0 1.0 Public domain dedication).

## Code availability

Code available from: https://github.com/proj-nuisance/nuisance

Archived code at time of publication: https://doi.org/10.5281/zenodo.3865441 ^21^.

License: Apache License 2.0

The aforementioned git repositories are also DataLad datasets that provide the complete computing environments used in the study (via git-annex), and contain full history of the analyses recorded in git commits history.
