# Supplementary material for: A new virtue of phantom MRI data: explaining variance in human participant data
Source: F1000Res. 2020 Sep 14;9:1131. [Version 1] doi: 10.12688/f1000research.24544.1 (PMC7783534; doi:10.12688/f1000research.24544.1)
Supplement: Peer review history

# Peer review: Reviewer response for version 1

**DOI:** 10.5256/f1000research.27077.r75970

**Published:** 2021-01-04

**Authors:** Jens Sommer

**Affiliations:** 1 Center for Mind, Brain and Behavior, Marburg and Justus-Liebig University Giessen, Philipps-University Marburg, Marburg, Germany; 2 Core Facility Brainimaging, Philipps-University Marburg, Marburg, Germany; 3 Psychiatry Department, Philipps-University Marburg, Marburg, Germany

The article describes in a very reasonable way how to estimate previously unexplained variance of MRI studies using data from the regular QA procedure of the radiology department. As the QA procedure is only run once a week the authors interpolate QA data or QA parameters to fit the dates of subject measurements.

 The original subject data is not available as the combination of individual measurement dates and mri data could disclose details about the subjects. But all of the procedures are available and described in sufficient detail, so it is possible to reproduce the study with local mri data.

 Right now I expect that the article could benefit if you would include more details about the QA procedure of the radiology department. Is the procedure described somewhere? If not, what gel phantom is used (FBIRN)? Do you use a mount for reproducible positioning? Has the phantom been replaced during the study period?

 Have there been any modifications or replacement of parts of the scanner hardware or software?

 Did all subjects undergo the same structural scan protocol, i.e. MPRAGE with identical TR, TI, TE, bandwidth? What kind of filters were applied? Was the volume of interest automatically or manually aligned?

 As you use some of the positional data for your analysis, it would also be nice to get an idea of the value ranges. Could you add a table or histogram to display the positional data?

 For the segmentation you used FAST from FSL 5.0.9 (described in Gosh *et al.*, your reference #13). My former colleague L. Eggert found FAST to be more sensitive to varying image quality than other algorithms (Eggert *et al.* ^1^). In his study he used FAST 4.1 from FSL 4.1.6.  I did not check the FAST version used in the container you used but according the FSL version history FSL 5.0.9 should have also included FAST 4.1.

 I would be glad if in a further analysis the statistics would still remain significant when grey matter volume is based on a different segmentation algorithm. But even without this analysis your idea of QA data usage is interesting.

Is the work clearly and accurately presented and does it cite the current literature?

Yes

If applicable, is the statistical analysis and its interpretation appropriate?

Yes

Are all the source data underlying the results available to ensure full reproducibility?

Partly

Is the study design appropriate and is the work technically sound?

Yes

Are the conclusions drawn adequately supported by the results?

Yes

Are sufficient details of methods and analysis provided to allow replication by others?

Partly

Reviewer Expertise:

QA procedures and improvement of MRI data in general

I confirm that I have read this submission and believe that I have an appropriate level of expertise to confirm that it is of an acceptable scientific standard, however I have significant reservations, as outlined above.

## References

[1] : Accuracy and reliability of automated gray matter segmentation pathways on real and simulated structural magnetic resonance images of the human brain. *PLoS One*.2012;7(9) : 10.1371/journal.pone.0045081 e45081 10.1371/journal.pone.0045081 23028771PMC3445568

# Peer review: Reviewer response for version 1

**DOI:** 10.5256/f1000research.27077.r75971

**Published:** 2020-12-21

**Authors:** Simon Duchesne

**Affiliations:** 1 Department of Radiology and Nuclear Medicine, Faculty of Medicine, Université Laval, Quebec City, QC, Canada

This is a great study addressing the subject of image quality and its influence on brain morphometric measurements. While most investigators assume that these measurements are indeed near infaillible, work of this kind serves to improve our collective ability at making these measurements ever more robust. 

 The authors have used a longitudinal series of AGAR phantom scans as well as human participants being recruited and scanned over the course of one year on a single (Siemens Healthcare Magnetom Prisma) machine. The statistical analysis is strong and demonstrate a possible effect of seasonal variations on the main outcome, namely grey matter segmentation.

 The paper has a lot of merit, and hopefully these comments will serve to further improve it.

 Major comments:

- The choice of the segmentation technique matters a lot. The inherent reliability of the technique may be the biggest inherent contributor to the variability in the results; further, this can be nonlinearly influenced by the scan characteristics. Speaking to the first point, the authors have chosen to use FSL, by which it is assumed they used the FAST technique. The authors are encouraged to read (and cite!) our recent publication on this topic (Dadar et al., NeuroImage 2020 ^1^ in which we compared various segmentation algorithms (FAST amongst them) on a human phantom dataset composed of repeat scans on multiple scanners of the same individual (albeit without correcting for time of day, SNR, and other metrics discussed here). In this work FAST was shown to be at the lower range of reliability, when compared to other publicly available techniques (an expedited review can be promised if the authors use our own technique!)(these open reviews allow for such clarity in reviewer motivations...). To speak to the second point, it becomes hard to dissociate without further experiments how a software segmentation tool may be more able (i.e. robust) than another at glossing over image quality variations in order to produce results with high reliability. Thus it may be that all of the quality metrics measured do influence the image; but the segmentation technique is able to remove these differences. Thus the agar phantom data is of greater importance here.
- Time of day seems indeed to matter a lot, possibly more than shown here. It would appear that most of the phantom scans were taken at the beginning of the week. It is rather typical to do these scans early on the first day of scans (i.e. Monday AM). Yet, multiple reports have shown that there is quite a lot of variability in image quality due to the scanner “warming up” after repeated activity, i.e. over the course of the day. Trefler and colleagues (NeuroImage 2016; ^2^) have done a nice study of this (you could cite them too). Thus in this study this effect may not have shown up in the phantom data, but could possibly affect the human results. Scattering phantom scans could address this issue.
- More generally, it is hard to draw conclusions on these effects with the choice of population. Studying GM in such a large number of different individuals on such a large age range is bound to be highly variable. It would be much better to focus on a subset of individuals (at the very least, only the young adults) and preferably only those with repeated measures, so that we can have a reasonable assumption that the scan-rescan variability should be very low. 

Minor comments:

- It is unclear what kind of interpolation between time points was done on the phantom data QC metric to estimate its value at the time of human scanning;
- The notion of IOPD is not quite clear. Does this mean some individuals were scanned 6 times? Otherwise, what goes into this metric? Patient position in the scanner is mentioned, but the position should always be the same (not like other techniques (e.g. chest x-ray) where you could have supine or prone, etc.)
- It should be discussed that the choice of “seasonal effect” as a variable on participants being recruited over a year masks realities that may bias the study irrespective of the seasonal effect on the MRI scanner itself. For example, a competing hypothesis could be that seasons generated a recruitment bias, with young participants being more available in the summer, while older participants in the winter. One would need to better define seasonal variations and what they entail in terms of signal characteristics that are related to the scanner - yet as independent as possible to the SNR. For example, seasonal effects could serve as a proxy of room temperature, which can be measured (and may even be retrospectively available, depending on the building information system). The latter would affect cooling of the main field - which in turn would affect the main B0 but possibly other sources of thermal noise in the RF apparati. Thus a metric such as room temperature would be better suited than a sinusoidal model of season. Unpublished data from a colleague (J. Doyon, Montreal Neurological Institute) have showed a distinct relationship between room temperature and phantom SNR.

Is the work clearly and accurately presented and does it cite the current literature?

Yes

If applicable, is the statistical analysis and its interpretation appropriate?

Yes

Are all the source data underlying the results available to ensure full reproducibility?

Yes

Is the study design appropriate and is the work technically sound?

Partly

Are the conclusions drawn adequately supported by the results?

Yes

Are sufficient details of methods and analysis provided to allow replication by others?

Partly

Reviewer Expertise:

Neuroimaging, imaging protocols, image processing, biomarkers, Alzheimer’s disease

I confirm that I have read this submission and believe that I have an appropriate level of expertise to state that I do not consider it to be of an acceptable scientific standard, for reasons outlined above.

## References

[1] : Reliability assessment of tissue classification algorithms for multi-center and multi-scanner data. *NeuroImage*.2020;217: 10.1016/j.neuroimage.2020.116928 10.1016/j.neuroimage.2020.116928 32413463

[2] : Impact of time-of-day on brain morphometric measures derived from T1-weighted magnetic resonance imaging. *Neuroimage*.133: 10.1016/j.neuroimage.2016.02.034 41-52 10.1016/j.neuroimage.2016.02.034 26921714PMC5602560

# Peer review: Reviewer response for version 1

**DOI:** 10.5256/f1000research.27077.r71693

**Published:** 2020-10-22

**Authors:** Blaise Frederick

**Affiliations:** 1 McLean Imaging Center, McLean Hospital, Belmont, MA, USA; 2 Harvard Medical School, Boston, MA, USA

This article explores the relationship between various factors (SNR on a QA phantom, patient positioning, time of year, etc) and the results of quantitation of anatomic MRI images.

 This is a solid piece of work, well executed and described, which demonstrates a useful method for reducing unexplained variance in MR data, in order to reveal underlying biological phenomena. The methods are very clearly described and reproducible, and all data is available for replication. I look forward to the companion paper on fMRI data. I would recommend accepting the paper as is (other than correcting Figure 2).

**Specific comments:**

 It would be worth explaining why different versions of dcm2niix were used for the phantom and human data. This is not a major issue - each dataset was processed consistently. It is a little odd however.

 Figure 2 shows a legend and y scale for a partial model fit, however no partial model fit appears in the graph, nor is there any mention of one in the text.

 While the correlation between gray matter volume and QA SNR is shown to be significant, it would be useful to mention the sign of the interaction - does apparent GM volume increase or decrease with increasing SNR? This would be an interesting thing to know.

 It would be a nice addition to the paper to see if there were any correlations between day of the week and any of the patient measures (not possible for the phantom data, since it was done once a week) to complement the Time-of-day and season metrics.

Is the work clearly and accurately presented and does it cite the current literature?

Yes

If applicable, is the statistical analysis and its interpretation appropriate?

Yes

Are all the source data underlying the results available to ensure full reproducibility?

Yes

Is the study design appropriate and is the work technically sound?

Yes

Are the conclusions drawn adequately supported by the results?

Yes

Are sufficient details of methods and analysis provided to allow replication by others?

Yes

Reviewer Expertise:

My area of expertise is fMRI and NIRS technique development, in particular methods for characterizing and interpreting physiological "noise" effects, and using them to quantify hemodynamics.

I confirm that I have read this submission and believe that I have an appropriate level of expertise to confirm that it is of an acceptable scientific standard.

# Peer review: Reviewer response for version 1

**DOI:** 10.5256/f1000research.27077.r71690

**Published:** 2020-10-06

**Authors:** Xiangrui Li

**Affiliations:** 1 Department of Psychology, The Center for Cognitive and Behavioral Brain Imaging, The Ohio State University, Columbus, OH, USA

This is an interesting topic and has great practical application for the MRI studies.

 My major concern is the conclusion about effect of variables, such as day time of acquisition, subject position, and SAR, on the brain tissue volume. It is easy to understand the potential effect of these variables on the phantom SNR, while it sounds a big jump to claim the effect on tissue volume. Even if other biological variables are included as confounds, the variability among participants is too large to be accounted by the potential minor effect of the phantom SNR.

 A better approach may be to test the effect of the phantom SNR on the volume of the same brain, but it seems there is not enough repeats for a single brain in the dataset.

Is the work clearly and accurately presented and does it cite the current literature?

Yes

If applicable, is the statistical analysis and its interpretation appropriate?

I cannot comment. A qualified statistician is required.

Are all the source data underlying the results available to ensure full reproducibility?

Yes

Is the study design appropriate and is the work technically sound?

Yes

Are the conclusions drawn adequately supported by the results?

Yes

Are sufficient details of methods and analysis provided to allow replication by others?

Yes

Reviewer Expertise:

MRI

I confirm that I have read this submission and believe that I have an appropriate level of expertise to confirm that it is of an acceptable scientific standard, however I have significant reservations, as outlined above.
